# Supplementary material for: A large dataset of white blood cells containing cell locations and types, along with segmented nuclei and cytoplasm
Source: Sci Rep. 2022 Jan 21;12:1123. doi: 10.1038/s41598-021-04426-x (PMC8782871; doi:10.1038/s41598-021-04426-x)
Supplement: Supplementary file 1 — Supplementary Information. [file 41598_2021_4426_MOESM1_ESM.pdf]

Appendix 1

In this section, we aim at explaining the image hash algorithms that we used for removing the duplicate images. The Average hash (A-hash) and Perceptual hash (P-hash) were employed to evaluate the similarity of the two cells. The Average hash has a very simple algorithm including the following steps:

- The image is resized to 8*8 pixels and converted to grayscale.
- The mean value of the pixels is computed: The pixels above this value take the value of 1, and the others take the value of zero. Indeed, we have a binary sequence with a length of 64 in row major order, for instance.
- The final hash is computed by converting the binary sequence to base 64.

The average hash is very fast and also robust against changes in brightness, contrast, color, size, and aspects of ratio. The perceptual hash which is more robust than the average hash includes the following steps:

- The image is resized to 32*32 pixels and converted to grayscale.
- The discrete cosine transform is computed and resized to 8*8 pixels.
- The mean value of the pixels is computed and similar to the average hash a binary sequence is obtained.
- The binary sequence is converted to base 64.

In order to compare two images, first, the desired hash is computed for each image. Then, the hamming distance between two hashes should be computed. After doing some experiments, we concluded that using the average and perceptual hash simultaneously provides better performance. We set two thresholds for the average and perceptual hash through trial and error (11 for A-hash and 14 for P-hash). Paired images with A-hash and P-hash distances less than tuned thresholds, are the same, and one of them should be removed.

Appendix 2

In this section we will explain more about the graph theory topics we used for removing duplicate images. Consider we have an undirected graph $G(V, E)$ that V and E are the sets of vertices and edges, respectively. A connected component is a subgraph $G'(V' \subseteq V, E' \subseteq E)$ in which there is a path between all pairs of nodes in V'. Both depth-first search (DFS) and breadth-first search (BFS) can be used to find all connected components of the graph [1]. We used BFS for this purpose. The pseudocode of the algorithm are as follows:

```
For each s ∈ V do
   If s is not visited
      Let Q be an empty queue.
      Let Connected_Component be an empty list.
      Mark s as visited.
      Q.enqueue(s)
      Connected_Component.append(s)
      While Q is not empty do
         v=Q.dequeue()
         for all neighbors w of v do
            if w is not visited
                    Q.enqueue(w)
                    Connected_Component.append(w)
                    Mark w as visited.
       Print(Connected_Component)
```

References

1. Skiena, S. S. *The algorithm design manual*. (Springer International Publishing, 2020)
